# Mapping the poultry insectome in and around broiler breeder pullet farms identifies new potential Dipteran vectors of *Histomonas meleagridis*

**DOI:** 10.1186/s13071-023-05833-x

**Published:** 2023-07-20

**Authors:** Maria Tereza Terra, Kenneth S. Macklin, Mark Burleson, Alan Jeon, John F. Beckmann, Ruediger Hauck

**Affiliations:** 1 Department of Poultry Science, Auburn University, Auburn, AL US; 2 Department of Poultry Science, Mississippi State University, Mississippi State, MS US; 3 Wayne Farms LLC, Oakwood, GA US; 4 Department of Entomology and Plant Pathology, Auburn University, Auburn, AL US; 5 Department of Pathobiology, Auburn University, Auburn, AL US

**Keywords:** Flies, Darkling beetle, Blackhead disease

## Abstract

**Background:**

*Histomonas meleagridis* can infect chickens and turkeys. It uses the eggs of the cecal worm *Heterakis gallinarum* as a vector and reservoir. Litter beetles (*Alphitobius diaperinus*) and other arthropod species have been implicated as potential vectors, but little information about other arthropod species as potential vectors is known.

**Methods:**

Four broiler breeder pullet farms were sampled every 4 months. On each farm, three types of traps were set inside and outside two houses. Trapped arthropod specimens were morphologically identified at order level and grouped into families/types when possible. Selected specimens from abundant types found both inside and outside barns were screened for *H. meleagridis* and *H. gallinarum* by qPCR.

**Results:**

A total of 4743 arthropod specimens were trapped. The three most frequently encountered orders were Diptera (38%), Coleoptera (17%), and Hymenoptera (7%). Three hundred seventeen discrete types were differentiated. More arthropods were trapped outside than inside. Alpha diversity was greater outside than inside but not significantly influenced by season. The composition of the arthropod populations, including the insectome, varied significantly between trap location and seasons. Up to 50% of litter beetles tested positive for *H. meleagridis* DNA 4 months after an observed histomonosis outbreak. Sporadically litter beetles were positive for *H. gallinarum* DNA. Thirteen further arthropod types were tested, and specimens of four Dipteran families tested positive for either one or both parasites.

**Conclusions:**

This study describes the insectome in and around broiler breeder pullet farms and identifies new potential vectors of *H. meleagridis* through qPCR. The results show a limited but present potential of arthropods, especially flies, to transmit histomonosis between farms.

**Graphical Abstract:**

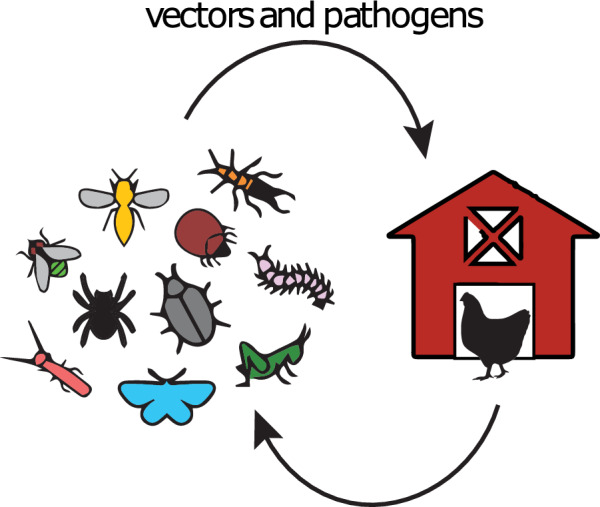

## Background

The protozoan parasite *Histomonas meleagridis* can cause a disease characterized by severe inflammation of the ceca and liver with high mortality of up to 90% in turkey flocks [1]. In chickens, the course of the disease is less severe, but it can still cause lesions in the ceca and liver and a significant drop in egg production [2, 3]. Currently, no drugs for prevention are available. Paromomycin, which is available in a few countries, can be given as a therapeutic drug, but the success of the treatment is uncertain [4]. Due to this situation, prevention of the introduction of the disease into poultry flocks is paramount, but transmission routes between flocks are still unknown.

In vitro cultivated histomonas cells survive only for a very short period outside their host [5], but the parasite can infect the gonads of the cecal worm *Heterakis gallinarum* and survive for long periods protected within the worm eggs [6–9]. Earthworms have long been identified as paratenic hosts of *H. meleagridis* and *H. gallinarum* [10]. However, we hypothesize that earthworms are less likely to be responsible for transmission between farms because of their limited mobility [11]. The role of arthropods as vectors for *H. meleagridis* and *H. gallinarum* is poorly explored. Blowflies (*Musca domestica* and *Lucilia* sp.) as well as several species of grasshoppers (*Bruneria brunnea*, *Camnula pellucida*, *Melanoplus mexicanus*, *Melanoplus packardii*, *Melanoplus bivittate*, and *Melanoplus femurubrum*) and sow bugs (*Porcellio scaber*) carried *Heterakis* eggs containing *Histomonas* stages for at least 4 days in their intestines after experimental exposure [12, 13], and *H. meleagridis* DNA has been detected in unspecified flies collected in poultry flocks [14].

More recently, it was shown that litter beetle (*Alphitobius diaperinus*) larvae, the lesser mealworm, can carry *H. meleagridis* after experimental exposure [15]. In addition, *H. meleagridis* and *H. gallinarum* DNA has been detected in litter beetles collected in poultry flocks, in some instances more than 1 year after a house was depopulated [16, 17]. Thus, litter beetles might be implicated as potential farm-to-farm vectors. However, when analyzing populations of litter beetles, we previously anecdotally noticed that they tended to stay in place and were not adept flyers [17]. More knowledge of litter beetle behavior and broader unbiased analysis of all potential flying arthropod vectors that might be responsible for transmission between farms would facilitate targeted control programs to lower the transmission of *H. meleagridis* among unexposed flocks/farms.

In the present investigation, we used extensive trapping in and around pullet houses to collect, identify, and characterize arthropod species (a vast majority being insects, hence “insectome”) in and around four unique broiler breeder pullet houses on two farms in Alabama over four visits. We then identified the most relevant potential vectors of *H. meleagridis* based on their prevalence inside and outside houses and conducted qPCR analysis for DNA of both *H. meleagridis* and *H. gallinarum*. In this analysis, we identify three new potential vectors which may be a vector of poultry pathogens due to their ability to fly in and out of pullet houses.

## Materials and methods

### Sampling

Four broiler breeder pullet farms in North Alabama belonging to two different companies were selected for this study. Two of them had a history of histomonosis. All farms had closed houses with dirt floors with tunnel ventilation and pine shavings as litter. Each house had about 20,000 pullets. Biosecurity measures included an anteroom with a disinfectant foot bath.

**Fig. 1:**
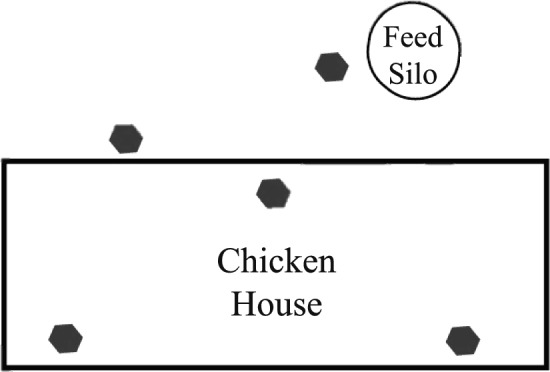
Floor plan showing the locations where three types of traps (gray hexagons) were set to capture arthropods in broiler breeder pullet farms. All types were set in all locations

During four visits in fall (October/November) 2020, spring (February/March) 2021, summer (June/July) 2021, and fall (October/November) 2021, one house on each farm, the same house on each visit, was sampled. Houses sampled at each visit and average outside temperatures and precipitation in the sampling periods are shown in Table 1. Three different trap types were placed in three locations inside the houses, as well as on the outside wall of the house, and at the feed silo (Fig. 1).

**Table 1:** Houses sampled at each visit and average temperatures and precipitation during sampling periods (data for Huntsville area from the National Weather Service)

Visit	Houses sampled	Average temperature (°C)	Average daily precipitation (mm)
October 2020	3 and 4	14.8	7.3
November 2020	1 and 2	11.3	0.1
February 2021	3 and 4	4.0	7.4
March 2021	1 and 2	15.7	16.4
June 2021	3 and 4	25.4	9.1
July 2021	1 and 2	28.3	0.3
October 2021	3 and 4	22.3	10.0
November 2021	1 and 2	12.4	2.4

**Fig. 2:**
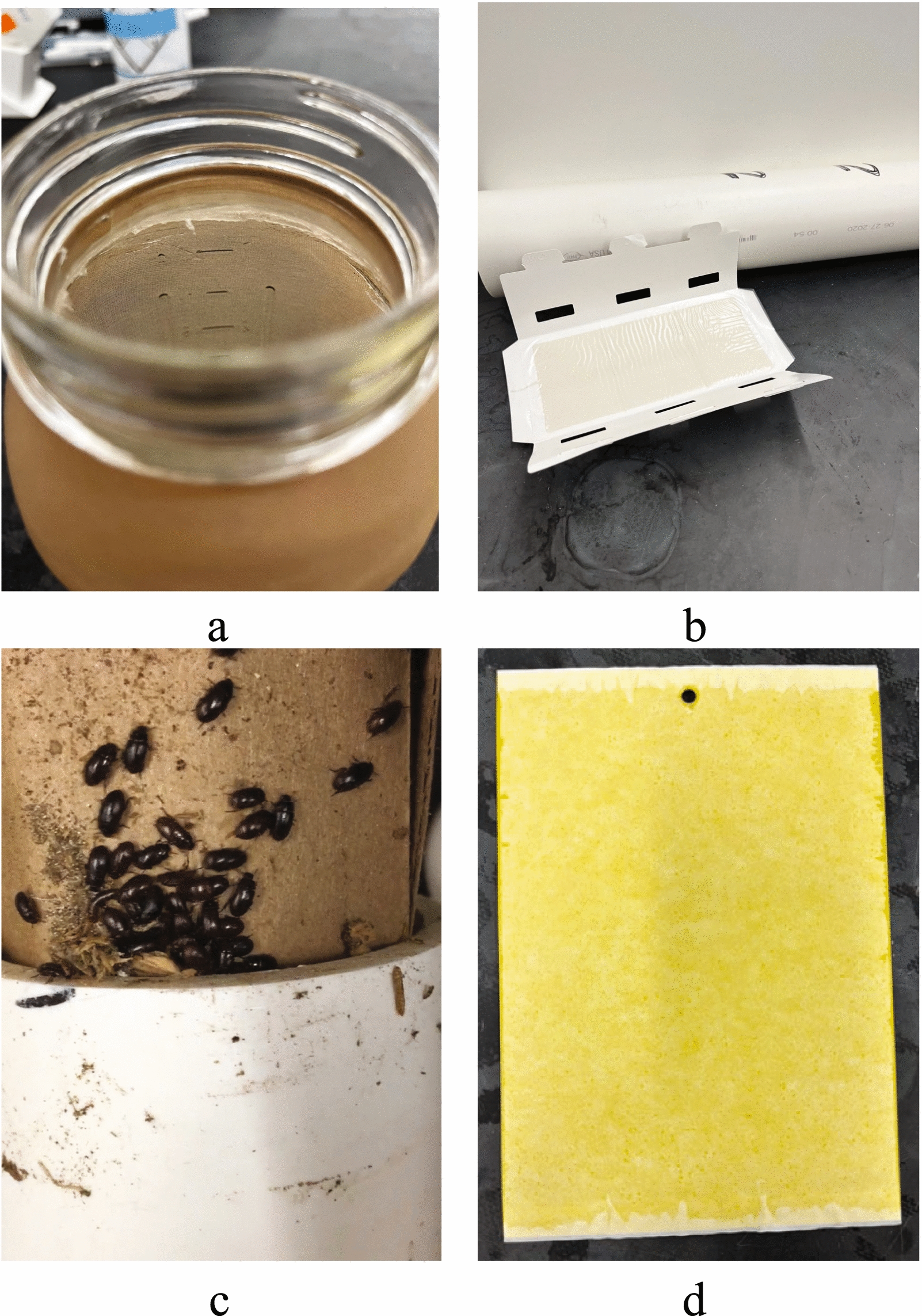
Traps used to capture arthropods in broiler breeder pullet farms. **a** Mason jars with bread and beer as baits covered with pantyhose for easier access to the trap by the invertebrates. Vaseline was used to prevent arthropods leaving the trap. **b** Pipe tube with a glue trap that was placed inside the tube. **c** Pipe tube with a piece of corrugated cardboard. **d** Yellow Sticky Gnat Trap to catch flying insects

The trap types were, first, 1-L mason jars with bread (White Sandwich Bread, Great Value by Walmart Rogers, AK) and lager beer (Anheuser-Busch, St. Louis, MO). The outside of the jar was covered with pantyhose for easier access for the invertebrates to climb the jar, while a ring of petroleum jelly (Unilever, London, UK) inside the jar helped to prevent the escape of trapped invertebrates (Fig. 2). The jars were placed on the ground protected from the chickens by upside-down buckets. Second, a piece of rolled-up corrugated cardboard measuring approximately 14 × 20 cm and a glue trap (Trapper Monitor and Insect, Bell Laboratories, Madison, WI) with a sticky area of 7.5 × 17.5 cm were placed inside at opposite ends of a PVC pipe with an inside diameter of 7.6 cm. The pipe was left on the ground. Third, yellow Sticky Gnat Traps measuring 15.2 × 20.3 cm (Kensizer, unknown location) were hung between 1.5 m and 2 m above ground. After one week, traps were collected and stored at −20 °C for later identification of the entrapped specimens.

### Morphological identification of arthropods

Trapped specimens were morphologically identified at the order level under a stereo microscope. Because morphological identification of species is not always possible, specimens were assigned to types, which likely represent one species, though we cannot be certain where specimens are deteriorated. Photos were taken to create an initial library of arthropod types, which were differentiated by notable morphological characteristics such as mouthpart types, wing count, halteres, the presence or absence of hair, color patterns of the wings, eye shape, etc., among other morphological markers. Specimens collected on the first visit were used as references for the following visits; often after the first set was counted new specimens in subsequent trappings were repeats of those originally identified. This fact suggested that we reached a reasonable coverage of the arthropod populations that were susceptible to our traps in the immediate area. Specimens collected during the second, third, and fourth samplings were counted, and, in case of types not observed before, new photos were taken and added to the library. Data were stored in a table containing type names, specimen counts, trap type, location of the trap, farm, company, and data of collection. After collection, specimens were identified to order, family, and species (if possible) by eye of trained taxonomic entomologists. All pictures were stored and the data retained.

### Analysis of alpha and beta diversity of populations

Shannon’s H and observed richness were calculated using the specnumber and diversity functions of the Vegan package in R [18, 19]. Pielou’s evenness was calculated as Shannon’s H divided by the log of the observed richness. Measures of alpha diversity were compared by two-way ANOVA with season and trap location as main effects with Tukey’s honestly significant difference test as a post hoc test using R [20]. For analysis of beta diversity, Bray-Curtis dissimilarity and Jaccard similarity coefficient were calculated using the vegdist function of Vegan. Principal component analysis (PCOA) and PERMANOVA were done for both measures using the cmdscale and adonis2 functions, respectively.

### Selection of samples for further investigation

If available, ten litter beetles per farm and time point were selected for testing for *H. meleagridis* and *H. gallinarum*. Other invertebrate types were selected for testing for *H. meleagridis* and *H. gallinarum* if > 20 specimens of the type had been collected and if specimens had been trapped inside as well as outside the houses on glue traps with the rationale being that species most likely to be main vectors of poultry pathogens would be abundant and capable of flying in and out of the pullet houses. Up to 10 specimens of each selected type per farm and time point were tested for the parasites. In addition, two specimens of the selected types were used for molecular species identification via PCR and Sanger sequencing.

### Investigation of samples

Histo-clear (National Diagnostics, Atlanta, GA) was used to recover specimens from the sticky traps to remove the glue as described by Butterwort et al. [21]. To guarantee a good DNA yield, arthropods were ground up with a handheld homogenizer (Hercuvan Lab Systems, Cambridge, UK) in a centrifugation tube before DNA extraction using the QIAamp DNA Mini Kit (Qiagen, Hilden, Germany) according to the manufacturer’s instructions.

*Histomonas meleagridis* and *H. gallinarum* DNA were detected by TaqMan probe-based qPCR. Reactions contained 10 μl of Forget-me-not qPCR master mix (Biotium, Fremont, CA), 6 μl of nuclease-free water, 2 μl of primermix (10 mM each), 1 μl of probe (2 mM), and 1 μl of DNA extracts. The primer/probe combination to detect *H. gallinarum* DNA was designed using Primer3 [22] based on *H. gallinarum* sequence MK122635 [16]. In silico evaluation by searching for sequences of primers and probe in the NCBI nucleotide collection yielded only the *H. gallinarum* sequence as a hit. The qPCR did not detect *Ascaridia galli* DNA and gave negative results with intestinal content of chickens known to be free of *H. gallinarum*. Primer sequences are given in Table 2. Reaction conditions were: 95 °C for 3 min, 40 cycles of 95 °C for 5 s, 60 °C for 30 s, and 72 °C for 30 s, followed by a final elongation at 72 °C for 10 min. Fluorescence was measured during the elongation step. Positive and negative controls were included in all runs.

**Table 2:** Primers used to detect *Histomonas meleagridis* and *Heterakis gallinarum* and to identify invertebrate types

Target	Forward primer	Reverse primer	Probe	Reference
*H. meleagridis*	CCG TGA TGT CCT TTA GAT GC ^a^	GAT CTT TTC AAA TTA GCT TTA AAT TAT TC	[6FAM] CTG CAC GCG CGC TAC AAT GTT AAA [BHQ1] ^b^	[38]
*H. gallinarum*	TGC ACG CAG TAT GGA CTA CG	CTG TAG GTT AGG CGC GAG AG	[6FAM] TGC AAC CGC TGT CTA TTT TTG GGG [BHQ1]	This study
Invertebrate 16S rRNA gene	TAR TYC AAC ATC GRG GTC	CYG TRC DAA GGT AGC ATA	No probe	[23]
Invertebrate COI gene	HCC HGA YAT RGC HTT YCC	TAT DGT RAT DGC HCC NG C	No probe	[23]

^a^All sequences are in 5’–3’ direction

^b^6-Carboxyfluorescein; Black Hole Quencher

For the molecular identification of arthropod species, two primer pairs targeting two universal genes were used. Chiar16SF/Chiar16SRF amplified approximately 348 base pairs (bp) of the 16S rRNA gene, and HexCOIF4/HexCOIR4 amplified approximately 260 bp of the cytochrome oxidase I gene (Table 2) [23]. Reactions contained 10 μl of Taq PCR Master Mix (Qiagen), 6 μl of nuclease-free water, 2 μl of primer mix (10 mM each), and 2 μl of DNA extracts. Reaction conditions were: 94 °C for 3 min, 35 cycles of 94 °C for 60 s, 44.9 °C for 60 s, and 72 °C for 60 s, followed by a final elongation at 72 °C for 5 min. PCR products were checked by agarose gel electrophoresis. Products of the Chiar16S PCR were gel purified using Qiaquick gel extraction kit (Qiagen). Products of the HexCOI PCR were purified using the Qiaquick PCR purification kit (Qiagen). Purified PCR products were submitted to the Massachusetts General Hospital DNA Core Facility for forward and reverse Sanger sequencing. The BLAST search algorithm [24] was used to compare the obtained sequence with sequences available from the National Center for Biotechnology Information (NCBI) database.

## Results

### Description of the insectome

**Fig. 3:**
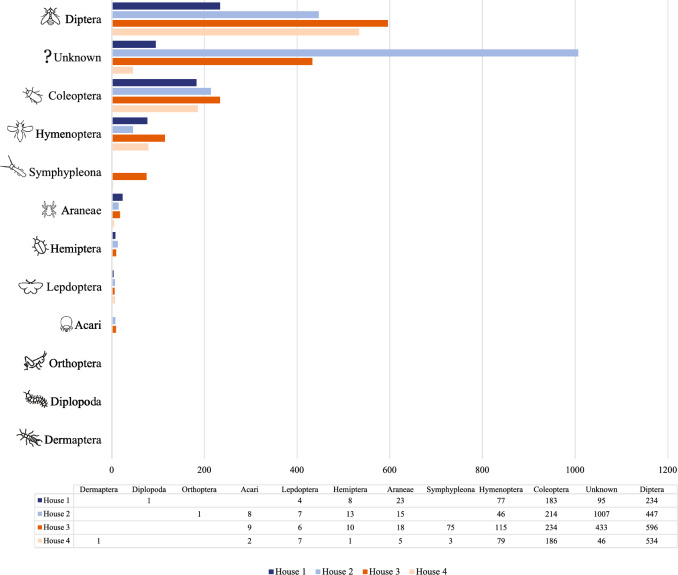
Number of arthropods of different taxonomic groups collected inside and outside four broiler breeder pullet houses. Order, subclass, or class is listed depending on what could be identified with confidence

**Fig. 4:**
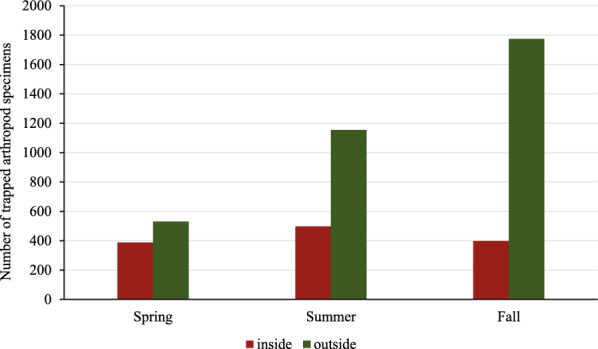
Comparison of the of total number of arthropods trapped in and outside broiler breeder pullet farms during visits 2 (spring), 3 (summer), and 4 (fall). Four houses were sampled per season

A total of 4743 arthropods were trapped and counted during the three visits in the spring, summer, and fall of 2021 (Table 3, Fig. 3). Of these, 1284 were trapped inside and 3459 outside the houses. Two hundred three arthropods (4%) were in the jars, 1097 (23%) were trapped in the pipes, and hanging glue traps caught 3443 flying arthropods (73%). Seasons influenced the differences in number of specimens trapped between inside and outside the houses (Fig. 4). Inside, seasonal differences were minor with a maximum in summer, while outside differences between the seasons were more noticeable with a maximum in fall. In addition to the adult stages, two different types of arthropod eggs were found in one pipe trap located outside the house after a visit in summer.

**Table 3:** Number of detected arthropods collected inside and outside four broiler breeder pullet houses with different trap types during three visits

Classification^a^	Inside			Outside			Total	Percentage (%)
	Jar	Pipe	Glue paper	Jar	Pipe	Glue paper		
Diptera	30	5	355	99	144	1178	1811	38.18
Unknown	17	1	82	34	39	1408	1581	33.33
Coleoptera	1	711	16	3	10	76	817	17.22
Hymenoptera	0	10	11	19	62	215	317	6.68
Symphypleona	0	0	0	0	78	0	78	1.64
Araneae	0	4	24	0	18	15	61	1.29
Hemiptera	0	0	2	0	0	30	32	0.67
Lepidoptera	0	2	11	0	1	10	24	0.51
Acari	0	0	1	0	9	9	19	0.40
Dermaptera	0	1	0	0	0	0	1	0.02
Diplopoda	0	0	0	0	1	0	1	0.02
Orthoptera	0	0	0	0	1	0	1	0.02
Total	48	734	502	155	363	2941	4743	100

^a^Order, subclass, or class is listed depending on what could be identified with confidence

**Fig. 5:**
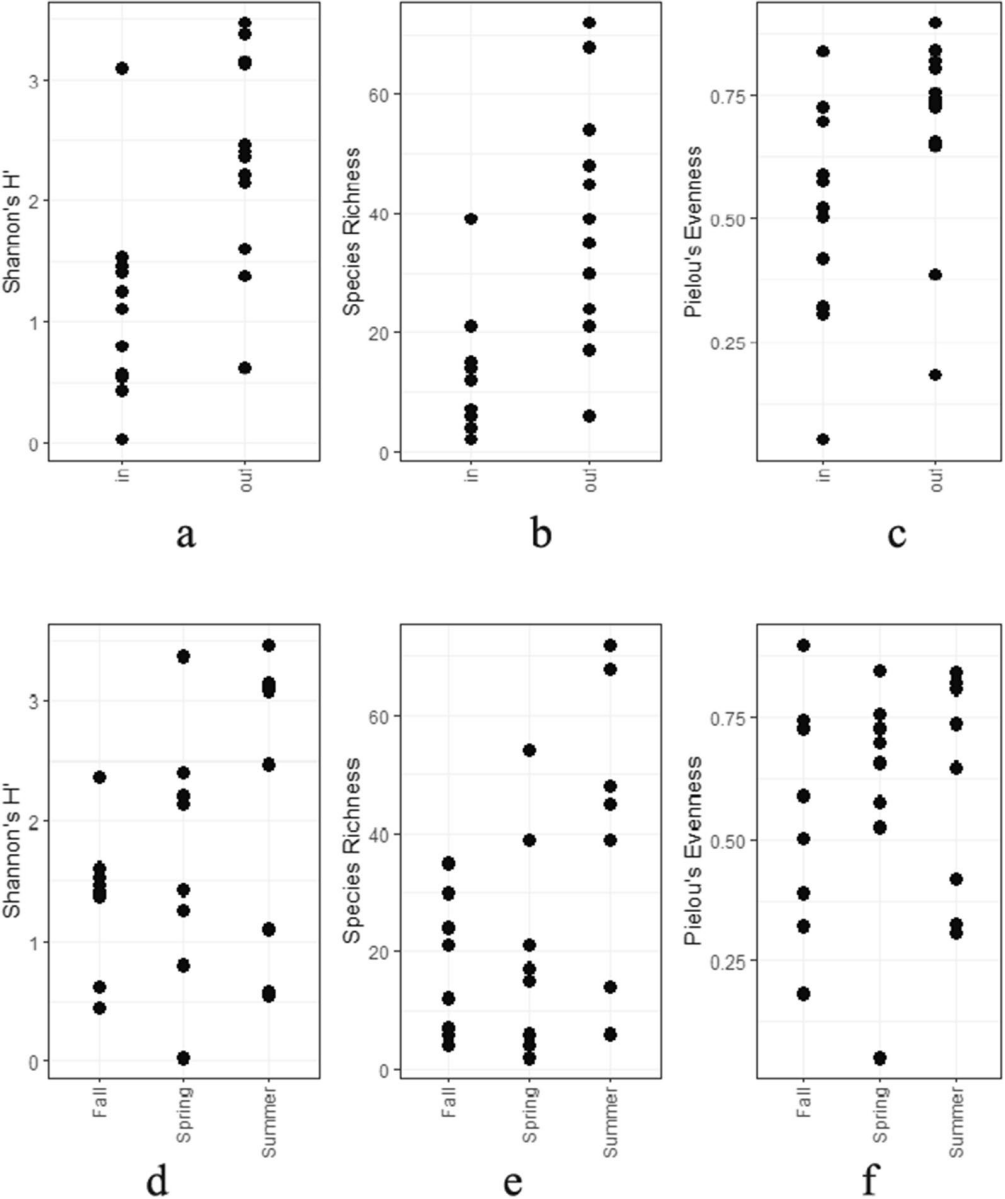
Alpha diversity of arthropod populations collected inside and outside broiler breeder pullet farms for 1 year measured by Shannon’s H (**a**, **d**), observed richness (**b**, **e**), and Pielou’s evenness (**c**, **f**). **a**–**c** Comparison between different seasons. **d**–**f** Comparison between the locations of the traps

Three hundred-seventeen different arthropod types were differentiated, and 3162 specimens could be attributed to 11 different orders. These were Acari (represented by mites), Araneae (represented by spiders and scorpions), Coleoptera (represented by beetles), Dermaptera (represented by earwigs), Diplopoda (represented by millipedes), Diptera (represented by flies), Hemiptera (represented by bedbugs and cicadas), Hymenoptera (represented by ants and wasps), Lepidoptera (represented by butterflies and moths), Orthoptera (represented by crickets and grasshoppers), and Symphypleona (represented by springtails). In addition, an unknown group accounting for 33% of the total arthropods counted was added, mostly consisting of arthropods that were too damaged to be classified.

The three most frequently encountered orders were Diptera (38%), Coleoptera (17%), and Hymenoptera (7%). Coleoptera was the only order that was far more frequently encountered inside the houses than outside. Seven hundred fourteen litter beetles were trapped, only three of them outside a house. In contrast, 67% of all arthropod types were collected outside the houses and another 11% inside as well as outside the houses. Thus, there was much more species richness outside than inside. Of the three litter beetles trapped outside, only one was found on a hanging glue trap and two litter beetles in mason jars. Inside the houses, 710 litter beetles were from the pipe traps and only one from a hanging glue trap.

### Analysis of alpha and beta diversity of populations

**Fig. 6:**
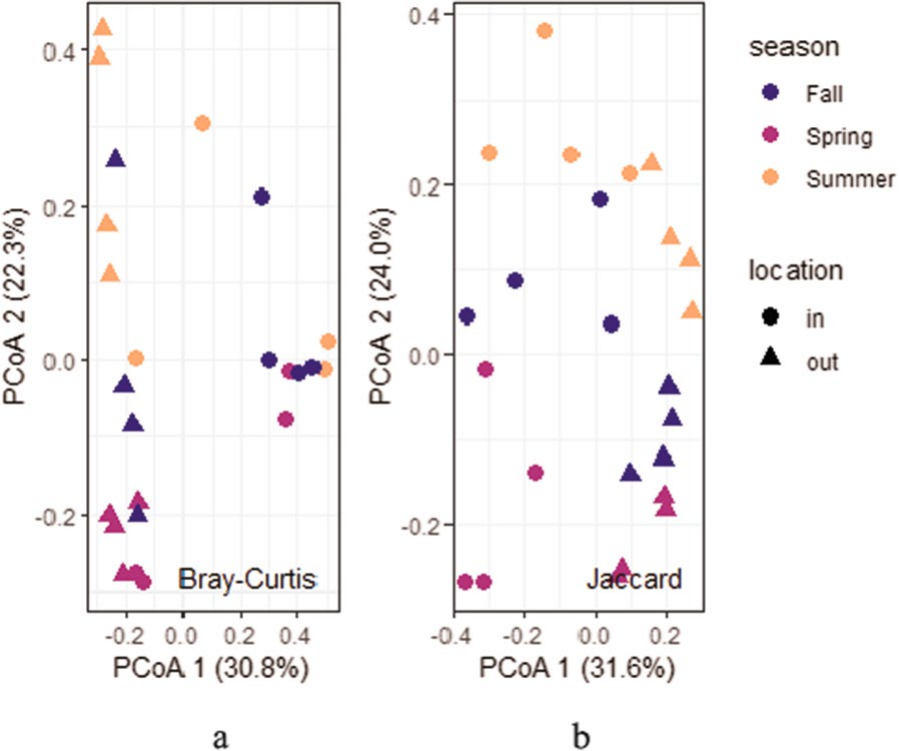
Principal component analysis of arthropods collected inside and outside broiler breeder pullet houses in different seasons based on Bray-Curtis dissimilarity (**a**) and Jaccard distance (**b**)

Alpha diversity as measured by Shannon’s H (*P* = 0.00143), observed richness (*P* = 0.00462), and Pielou’s evenness (*P* = 0.036) was significantly higher inside houses than outside houses (Fig. 5a–c). Alpha diversity did not significantly differ between seasons (Fig. 5d–e). There was no significant interaction between the location of the trap and the season. PCOA (Fig. 6) and PERMANOVA showed a significant influence of trap location (*P* < 0.001 based on Bray-Curtis dissimilarity and based on Jaccard similarity coefficient) and season (*P* = 0.039 based on Bray-Curtis dissimilarity and *P* < 0.001 based on Jaccard similarity coefficient) on the composition of the arthropod populations. The farm had no significant influence (*P* = 0.835 based on Bray-Curtis dissimilarity and *P* = 0.796 based on Jaccard similarity coefficient).

### Investigation of samples for *H. gallinarum* and *H. meleagridis*

**Fig. 7:**
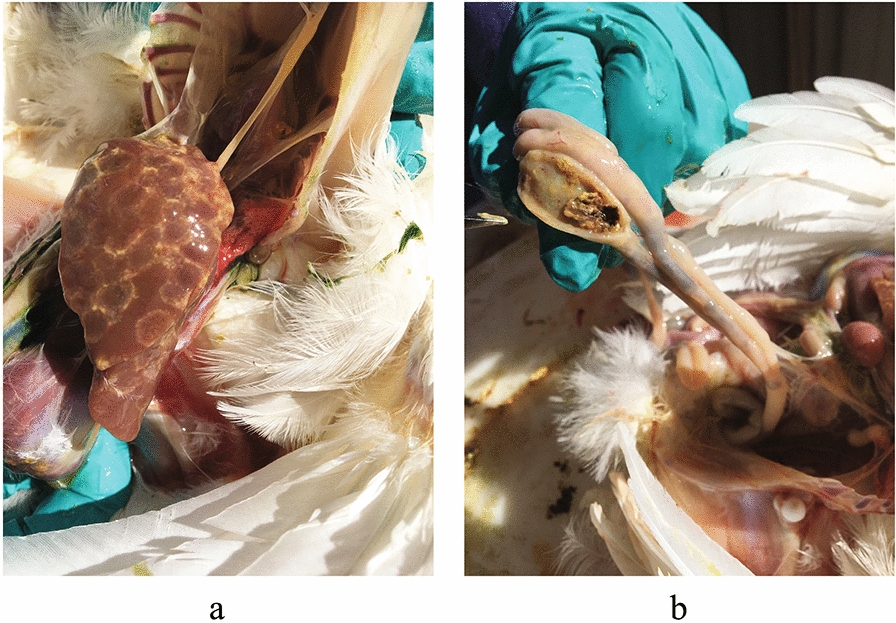
Lesions observed during a histomoniasis (blackhead) outbreak in one of the broiler breeder pullet farms where arthropods were collected. **a** Necrotic targetoid areas in the liver **b** Caseous cecal content

One of the four farms investigated had a histomoniasis outbreak during the second visit. In the liver were focal to multifocal necrotic targetoid areas across the organ. In the ceca, no cecal worms were found; however, alterations in the consistency of the content were seen from being frothy in mild cases to caseous in more stricken birds (Fig. 7). The diagnosis was confirmed by qPCR.

**Fig. 8:**
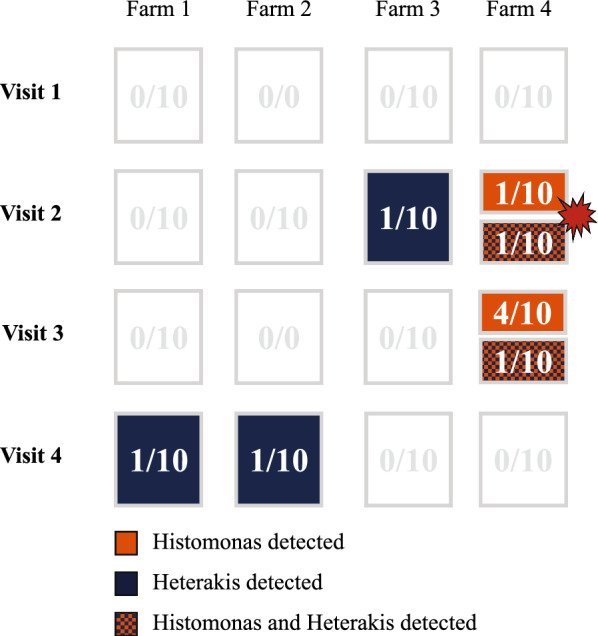
Representative scheme of location and time when litter beetles collected at four broiler breeder pullet farms for 1 year were positive for *Histomonas meleagridis* and/or *Heterakis gallinarum* by PCR. The red balloon shows the time point when the histomoniasis outbreak occurred. Numbers in the boxes show the number of positive samples and the number of investigated samples. Positive samples for *H. meleagridis* are represented in orange boxes. Positive samples for *H. gallinarum* are represented in blue boxes. Positive samples for both are represented in orange and blue boxes

In total, 142 litter beetles were tested for *H. gallinarum* and *H. meleagridis*. Of these, three litter beetles tested positive for only *Heterakis*, five litter beetles were positive for only *Histomonas*, and two litter beetles tested positive for both parasites. Out of these 10 positive litter beetles, 7 were from the same farm where the outbreak occurred. Two litter beetles were collected at the time of the outbreak, and five were collected on visit 3, which was 4 months later (Fig. 8). Thus, during an outbreak an estimate of litter beetles carrying *Histomonas* DNA is 20%, while 4 months later 50% tested positive for *Histomonas* DNA.

**Fig. 9:**
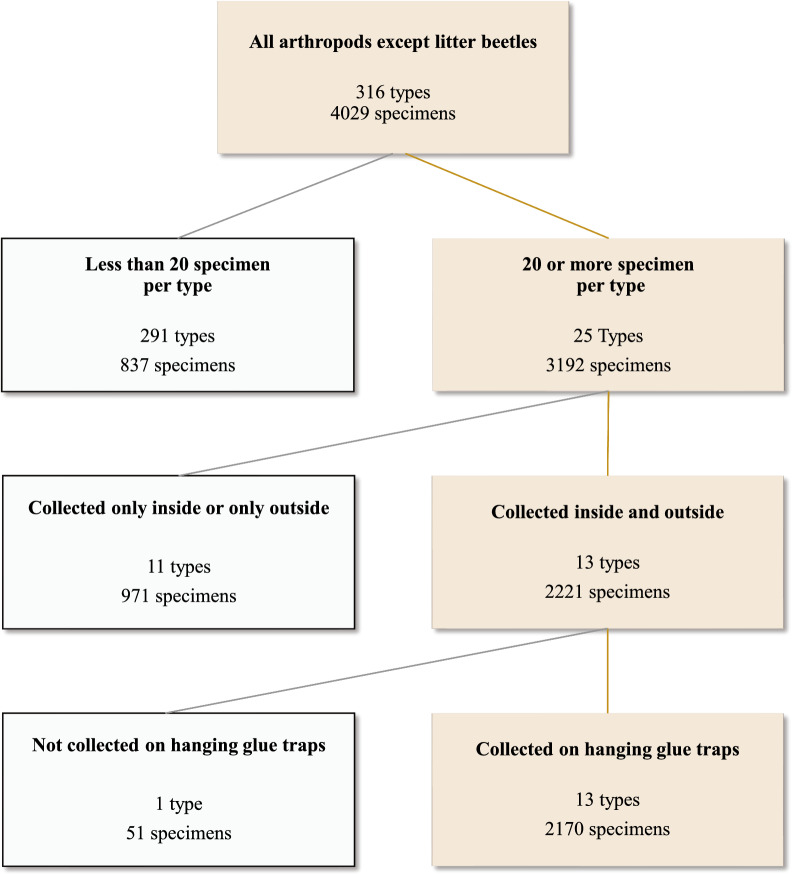
Decision tree showing how arthropod types were selected for further testing by *Histomonas* and *Heterakis* qPCR. Gray boxes on the left show the numbers of types and specimens that did not meet inclusion criteria; light brown boxes on the right show the numbers of types and specimens that met inclusion criteria

Thirty-six invertebrate types (11%) were trapped inside as well as outside the houses, and 13 of these were represented by 20 or more specimens. Two thousand one hundred forty-three (46%) specimens belonged to types that were caught inside and outside on glue traps (Fig. 9). However, 927 specimens belonged to type UK14, which was detected only at one visit to a single farm. Most types were detected at three or four farms and in spring, summer, and fall (Table 4).

**Table 4:** Arthropod types of which 20 or more specimens were trapped and which were detected inside as well as outside poultry houses on glue traps

Type	Order	Family	Genus	Species	Inside^a^			Outside^a^			Houses^b^
					Sp	Sum	Fall	Sp	Sum	Fall	
F21	Diptera	*Muscidae*	*Musca*	–	0	0	6	0	1	3	4
B5	Coleoptera	*Silvanidae*	*Ahasverus*	*advena*	0	10	1	0	2	16	3
BG^c^	Diptera	*Dolichopodidae*	*Condylostylus*	–	0	1	2	0	0	209	4
F1^c^	Diptera	*Sciaridae*	*Bradysia*	–	1	20	5	0	28	34	4
F4	Diptera	*Phoridae*	*Megaselia*	*scalaris*	0	0	0	0	0	0	4
F5	Diptera	*Sciaridae*	*-*	–	0	2	7	0	2	9	4
FB	Diptera	*Muscidae*	*Musca*	–	0	12	1	0	32	19	2
FF	Diptera	*Drosophilidae*	*Drosophila*	*repleta / tripunctata*	0	0	16	0	0	4	2
FM	Diptera	*Cecidomyiidae*	*Catocha*	–	7	0	1	0	82	0	4
TB^c^	Diptera	*Sphaeroceridaa*	–	–	0	9	1	0	29	21	4
UK14	Hemiptera	*Afidídeos*	*Capitophorus*	–	20	19	27	0	7	67	1
UK2	Diptera	*Psychodidae*	*Psychoda*	–	0	1	0	0	0	0	4
YB^c^	Diptera	*Muscidae*	*Musca*	–	66	0	1	0	10	0	4

^a^Number of specimens inside or outside of houses/in a given season (Sp. = spring, Sum. = summer)

^b^Number of houses in or near which the type was detected

^c^Positive samples for *Histomonas meleagridis* or *Heterakis gallinarum*

**Fig. 10:**
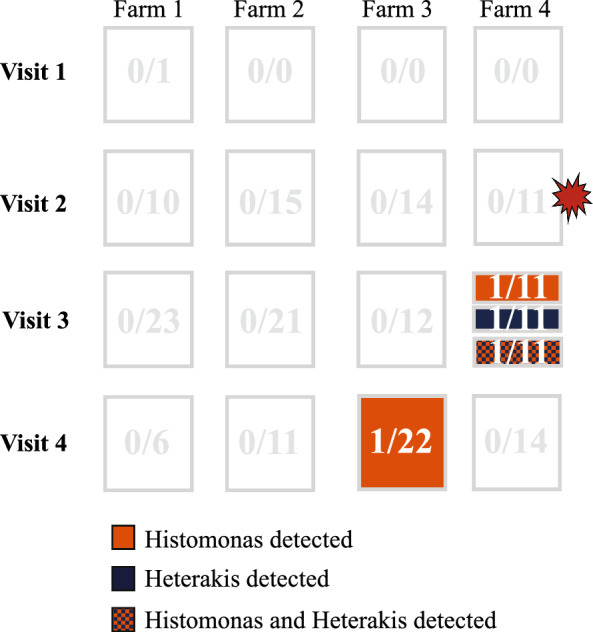
Representative scheme of the location and time when PCR samples of 11 different types of flies, one beetle type, and one hymenoptera collected at broiler breeder pullet farms for 1 year were positive for *Histomonas meleagridis* and or *Heterakis gallinarum*. The red balloon shows the time point when the histomoniasis outbreak occurred. Positive samples for *H. meleagridis* are represented in orange boxes. Positive samples for *H. gallinarum* are represented in blue boxes. Positive samples for both are represented in orange and blue boxes

These types were selected for further investigation, and a total of 171 specimens were tested. Of these, two flies were positive for *Histomonas* DNA, one was positive for *Heterakis* DNA, and one fly for both. Three flies were collected at the farm that had the outbreak 4 months after it occurred, and one was collected at farm 3 (Fig. 10). Two flies that were positive for *Histomonas* were trapped outside. The four types with *Histomonas*-positive specimens represented 17.2% of the total arthropod specimens collected. All four types were detected at all four houses, and, with one exception, in spring, summer, and fall (Table 4).

### Sanger sequencing identification of arthropods

Partial 16S rRNA and COI genes of the 13 selected types were sequenced. For each type, two specimens were investigated. Sequences were compared with published sequences in the NCBI database. Identification was based on the similarity score. If several species had similar scores or if results of the two genes were not in agreement, a combination of scores and the reference photos was used for identification. The results are summarized in Table 4. All 14 types were identified at the family level. Twelve were identified at the genus level, and four were confidently identified at the species level. Twelve types were from the order Diptera, and one each was from Coleoptera and Hemiptera. *Musca* was the most common genus identified with three different types. The other genera found were *Ahasverus*, *Condylostylus*, *Bradysia*, *Megaselia*, *Drosophila*, *Capitophorus*, and *Psychoda*. The taxa that were positive for either for *H. gallinarum* or *H. meleagridis* were *Condylostylus* (long legged flies), *Musca* (house flies), *Bradysia* (fungus gnats), and *Sphaeroceridae* (small dung flies)*.*

## Discussion

This is the first comprehensive detailed investigation of arthropod diversity inside and outside broiler breeder pullet farms. Even though pest control is an important component of a successful biosecurity program, little attention has been given to the invertebrates that can be found in and around farms and diseases they might transmit. Studies investigating their risk potential are still rare. The exception is the litter beetle with a number of papers evaluating its role as a reservoir of different bacteria and viruses [25–28]. While litter beetles undoubtedly make conditions less sanitary and potentially transmit poultry pathogens, multiple data points from this study call into question the notion that they are a main vector spreading histomonosis farm to farm. First, our data clearly indicate that litter beetles rarely leave the barn; only three beetles were detected outside. Second, litter beetles rarely fly; only two beetles were detected on above-ground traps in contrast to the vast majority caught on the ground. These data do not rule them out as vectors, but they do call into question whether they are the main or most important vector.

Three different trap types were used in the present study. The jars did not trap as many arthropods as the other types, with the discrepancy being greater inside the houses (Table 3). In addition, collecting the arthropods from the 1-week-old bread and beer made sample recovery almost impossible. In comparison, the pipes with cardboard worked very well for capturing litter beetles and should be considered the method of choice in future studies. The glue traps were suitable for flying arthropods.

Almost 5000 arthropods were counted, and 317 different types were identified. Eighty-three percent of specimens that could be attributed to an order were either Diptera or Coleoptera, two orders that were already described as being prevalent in poultry production [28, 29]. However, the remaining 17% show that the invertebrate population around poultry houses is more diverse.

Analysis of alpha and beta diversity showed significant differences between traps inside and outside houses. This was illustrated by the result that only 10% of types were trapped inside as well as outside the houses. This limited overlap of arthropod populations shows that only few arthropod species could act as long-range vectors carrying pathogens from one poultry farm to another. Seasonal differences in the arthropod population not only outside the houses but more surprisingly also inside the houses further might limit the risk of arthropod-vectored transmission to certain times of the year. However, the types that we identified as of interest based on their detection inside as well as outside the houses and their ability to fly were mostly detected on all farms and during several seasons.

One specific aim of the investigation was to test samples for *H. gallinarum* and *H. meleagridis*. The persistence of their DNA in litter beetles and in the environment for an extended time has already described in previous studies [17, 30]. Compared to a previous study investigating litter beetles in poultry houses that had been empty for months [17], the detection of the DNA was more sporadic in the present investigation. One possible explanation might be a higher turnover of the beetles in the field compared to empty houses. Beetles will reproduce faster with warmer temperatures and better feed supply [31]. On the other hand, birds, especially feed-restricted broiler breeder pullets, will eat beetles, and use of pesticides will reduce the numbers. In two visits to one of the farms, no beetles were recovered. However, insecticides can only be applied between flocks. Thus, infestations often return when birds are present.

Sanger sequencing identified three genera that were already correlated with poultry production. These were *Musca* [32], *Ahasverus* [27], and *Drosophila* [29]. A survey done in Brazil evaluated the prevalence of Diptera species on layer farm for 2 years and identified that *D. repleta* (identified in our study) and *M. domestica* represented 99.47% of the dipterans [33]. On the other hand, *Bradysia* and *Condylostylus* have not been previously described at chicken farms nor have they ever been shown to test positive for *Histomonas* DNA until this study, to our knowledge. Bradysia has about ~ 400 characterized species with a worldwide distribution; they prefer to live in humid environments [34]. Condylosylus has about 314 described species worldwide with most being neotropical [35].

In Europe, the number of histomonosis cases has a small peak during warmer months [14, 36]. The outbreak we observed occurred during a colder period in spring with average temperatures of about 4 °C. The impact of weather conditions on the insectome is clear, but further studies need to investigate whether there is a connection, direct or indirect, with histomonosis outbreaks.

One major limitation of the study is the use of qPCR to detect the parasites. qPCR detects DNA, and conclusions should not be drawn about transmission of live parasites from qPCR-derived data. Thus, no conclusion about the infectivity of the positive arthropods is possible. While *Heterakis* eggs can remain infective in the environment for a long time, *Histomonas* does not [5], and the postulated existence of resistant stages remains unproven [37]. The other limitations are that that some relevant arthropod species could not be trapped with the traps we used and that populations in other regions might be different from the ones observed in North Alabama. On the other hand, our study is important because qPCR identification of *Histomonas* in arthropods that fly inside and outside of pullet barns is the first step toward identifying vectors.

## Conclusion

This study described, for the first time to our knowledge, a robust analysis of arthropod populations in and around broiler breeder pullet farms and identified new potential vectors of *H. meleagridis* through qPCR. The results show a limited but present potential of arthropods, especially flies, to transmit histomonosis between farms.

## Data Availability

The authors declare that all the data related to this study are cited in the text.
